# *Cistus monspeliensis* L. as a potential species for rehabilitation of soils with multielemental contamination under Mediterranean conditions

**DOI:** 10.1007/s11356-017-0957-3

**Published:** 2017-12-16

**Authors:** Daniel Arenas-Lago, Erika S. Santos, Luisa C. Carvalho, Maria Manuela Abreu, Maria Luisa Andrade

**Affiliations:** 1 Department of Plant Biology and Soil Sciences, Universidad de Vigo, Lagoas Marcosende, 36310 Vigo, Spain; 2 Institute of Environmental Sciences (CML), Leiden University, P. O. Box 9518, 2300 RA Leiden, The Netherlands; 3 Instituto Superior de Agronomia, Linking Landscape, Environment, Agriculture and Food Research Center (LEAF), Universidade de Lisboa, Lisbon, Portugal

**Keywords:** Antioxidative enzymes, Glutathione-ascorbate cycle, Metal(loid)s, Mine soils, Soil rehabilitation

## Abstract

The Iberian Pyrite Belt (IPB; SW of the Iberian Peninsula) is one of the most important volcanogenic massive sulphide ore deposits in the world. *Cistus monspeliensis* L. is a native woody shrub that grows spontaneously in non-contaminated soils as well as in soils with multielemental contamination from the IPB. In this study, different ecophysiological parameters of *C. monspeliensis* growing in soils with different levels of metal(loid)s were evaluated to assess the potential of this species for revegetation of degraded areas. Composite samples of plants and rhizosphere soils were sampled in São Domingos and Lousal mines and in a reference area without soil contamination (Pomarão, Portugal) (Portuguese sector of IPB). Classical characterisation of the soils and quantification of their total and available metal(loid) concentrations were done. Multielemental concentration was determined in plants (shoots and roots). Ecophysiological parameters were also determined in shoots: concentrations of pigments (chlorophylls, anthocyanins and carotenoids), antioxidants (glutathione and ascorbate) and hydrogen peroxide as well as activities of several antioxidative enzymes. Although mining soils present high total concentrations of potentially hazardous elements, their available fractions were low and similar among studied areas. Soil pH as well as concentrations of extractable P, total concentrations of As, Cd and Ni and concentrations of Cu, Cr, Ni, Pb and Sb in the soil available fraction differentiate the studied areas. Only concentrations of Cd, Pb and Sb in roots and shoots were explained by the concentrations of the same elements in the soil available fraction. Although the majority of elements were translocated from roots to shoots, the shoots concentrations were below the toxic values for domestic animals and only As, Mn and Zn reached phytotoxic concentrations. Ecophysiological parameters were similar independently of the studied area. Due to its adaptability, tolerance and standard plant features, *C. monspeliensis* is a good choice for rehabilitation of soils with multielemental contamination under similar climatic characteristics.

## Introduction

Iberian Pyrite Belt (IPB; SW of the Iberian Peninsula) is one of the most important volcanogenic massive sulphide ore deposits in the world (Tornos 2006). In the mines from the Portuguese sector of IPB (PIPB), as a result of open cast and underground mining operations, and lack of environmental management practices, large amounts of waste materials with high contents of metal(loid)s were exposed to weathering conditions and pedogenesis (Matos and Martins 2006; Santos et al. 2017) leading to the formation of incipient soils (Spolic Technosols; IUSS Working Group WRB 2015). These soils, as the mine wastes, have usually significant chemical and physical limitations to plant development, such as low pH and organic matter content, unfavourable texture and structure and high total concentrations of metal(loid)s (Abreu and Magalhães 2009; Santos et al. 2017). Additionally, the oxidation of the metallic sulphides from the PIPB mine spoils results in acid mine drainage generation with the consequent release and leaching of significant amounts of metal(loid)s leading to the contamination, alteration and destruction of the adjacent ecosystems (Abreu et al. 2010; Ferreira da Silva et al. 2005; Santos et al. 2016a, 2017). These extreme conditions of the soils and mine wastes from the PIPB inhibit/reduce the growth of the spontaneous vegetation cover contributing to the increase of hydric and wind erosion and, consequently, the spreading of the contamination (Abreu and Magalhães 2009; Santos et al. 2016a).

Nevertheless, it is not uncommon that Technosols developed on some types of mining wastes from the PIPB are colonised by autochthonous plant species (e.g. genus *Cistus*, *Lavandula* and *Erica*), which grow spontaneously without any visual signs of toxicity despite the multielemental contamination in soils and relative high concentrations of some metal(loid)s in their roots and shoots (Abreu et al. 2008, 2012a, b; Batista et al. 2017; Freitas et al. 2004; Márquez-García and Córdoba 2009; Pérez-López et al. 2014; Santos et al. 2012, 2014, 2016b, c). These plants provide important contributions for natural rehabilitation of the contaminated soils, decreasing the elements spreading by leaching and erosion (Abreu and Magalhães 2009; Tordoff et al. 2000). Also, the establishment of a self-sustaining vegetation, principally with pioneer species, contributes to the ecological succession. In general, several *Cistus* species growing in soils developed on mine wastes from the IPB present adequate ecological behaviours to the rehabilitation of these materials (Abreu et al. 2012a, b; Batista et al. 2017; Alvarenga et al. 2004; Freitas et al. 2004; Santos et al. 2009, 2012, 2014).

The uptake and accumulation of high contents of metal(loid)s in the plant tissues may often cause oxidative stress, resulting in an increase of reactive oxygen species (ROS) and, consequently, a significant damage at the physiological and cellular levels. In order to withstand oxidative stress, plants have developed several ecophysiological mechanisms/strategies of tolerance such as, the decrease of elements absorption and/or translocation to the aboveground organs, the intracellular sequestration as well as stimulation of the activities of antioxidative enzymes and production of non-enzymatic compounds (e.g. glutathione, ascorbic acid, carotenoids) involved in the scavenging of ROS (Abreu et al. 2014; Caverzan et al. 2012; Hall 2002; Márquez-García and Córdoba 2009; Pang et al. 2003; Rossini Oliva et al. 2009a; Santos et al. 2009, 2016c). Many plant species growing in soils with multielemental contamination from the IPB mining areas have developed these strategies (Abreu et al. 2008, 2012a, b; Pérez-López et al. 2014; Rossini Oliva et al. 2009a, b; Santos et al. 2012, 2014, 2016c).

*Cistus monspeliensis* L. is an autochthonous species, well adapted to Mediterranean conditions (Correia 2002; Sánchez-Blanco et al. 2002) and to less acid soils (Núñez-Olivera et al. 1995) that grows spontaneously in several mining areas from the IPB, including São Domingos and Lousal, as well as in uncontaminated areas in the vicinity of these mines. However, little information exists about the ecophysiological features of this species and its potential for natural rehabilitation of contaminated soils.

In this study, the ecophysiological features of *C. monspeliensis* growing in soils with multielemental contamination (São Domingos and Lousal mine areas) and in a reference area without soil contamination and same climatic conditions (Pomarão) were compared in order to evaluate the potential of this species for the revegetation of soils with multielemental contamination. For this, the metal(loid) storage capacity in roots and shoots and elements translocation to shoots as well as different antioxidative enzymes and antioxidant molecules associated to oxidative stress were evaluated.

## Material and methods

### Study areas and sampling

This study was carried out in two different abandoned mining areas from PIPB, São Domingos (Datum WGS84: 37.6683, − 7.4939) and Lousal (Datum WGS84: 38.0369, − 8.4278), and in a reference area without soil contamination located about 18 km to the South of the São Domingos mine (Datum WGS84 37.5949, − 7,5361) (near Pomarão village). According to Thornthwaite classification, the climate of these areas is semiarid mesothermic (average of the minimum air temperatures: 5–16 °C; average of the maximum air temperatures: 14–33 °C; and the average of the annual precipitation: 548 mm; Climate normals 1981–2010, Beja, IPMA 2016). The soil moisture and soil temperature regimes are considered xeric and thermic, respectively (SSS 1999).

São Domingos mine was exploited in two periods: before and during the Roman period for Ag, Au and Cu, and later, from the middle of the nineteenth century until 1960 for massive sulphides and *gossan*, mainly for Cu, Zn and S extraction (Matos and Martins 2006; Quental et al. 2002). The Lousal mine was exploited between 1900 and 1988, mainly for pyrite (Matos and Martins 2006). Mining operations in São Domingos and Lousal caused the degradation of the natural landscape including soils and superficial waters. In both mine areas, high volumes of wastes were disposed irregularly affecting large areas and generating acid mine drainage. Bare wastes and some contaminated soils are, in raining periods, subject to significant hydric erosion (Abreu et al. 2010; Ferreira da Silva et al. 2005; Matos and Martins 2006; Quental et al. 2002).

In the three studied areas, different sampling zones were selected to include representative soils where *C. monspeliensis* grows (five in São Domingos mine; four in Lousal mine and three in Pomarão). Soils in São Domingos and Lousal are thin and were developed over spoils, composed mainly by gossaneous materials and host rocks (Spolic Toxic Technosols) (IUSS Working Group WRB 2015), or developed on schists and greywackes (Lithic Leptosols) (IUSS Working Group WRB 2015) and influenced by particulate materials and/or acid mine drainage from adjacent tailings. In Pomarão, the soils (Lithic Leptosols) (IUSS Working Group WRB 2015) were developed on schists and greywackes belonging to the Flysch Group of the Baixo Alentejo (Oliveira et al. 1984).

*Cistus monspeliensis* grows in the sampling zones usually forming isolated groups of 5–10 individuals occupying a surface cover of ca. 3–10 m^2^. Composite samples of shoots (composed of leaves and twigs) and roots were collected in each sampling zone and in at least three different adult plants with height ranging from 1.0 to 1.5 m. In each zone, soil samples from the surface horizon (0–20 cm depth and ≈ 3 kg of homogenate soil) were collected surrounding the rhizosphere system of all harvested plant, obtaining a composite sample. Sampling was performed in spring, after the rain period.

### Chemical analysis of soils and plants

Soil samples were air-dried, sieved through a 2-mm mesh and homogenised. These samples (fraction < 2 mm) were analysed for (Póvoas and Barral 1992): pH in water suspension (1:2.5 *m*/*V*), total organic C by wet combustion, extractable P and K using the Egner–Riehm method (LV ST ZM 82–97), where 0.04 M calcium lactate extraction is used as an extracting agent being acidified by hydrochloric acid up to pH 3.5–3.7 (Egnér et al. 1960), and total N by the Kjeldahl method (Kjeldahl 1883). The multielemental total concentration of the soils was determined by instrumental neutron activation analysis and inductively coupled plasma after acid digestion with perchloric, nitric, hydrochloric and hydrofluoric acids (Activation Laboratories 2015a). The multielemental concentration of the soil in the available fraction was determined by inductively coupled plasma mass spectrometry (ICP-MS) and inductively coupled plasma optical emission spectrometry (Activation Laboratories 2015b), after extraction by the rhizosphere-based method (Feng et al. 2005).

Plants were washed with tap water followed by distilled water, and the roots were cut and sonicated in distilled water in an ultrasound bath for 30 min. The plant samples were dried at 40 °C, homogenised and finely ground. Multielemental chemical analysis of the shoot and root samples was carried out by ICP-MS, after reducing the samples to ashes at 475 °C followed by digestion with nitric acid (Activation Laboratories 2015c). Quality control of the elemental analysis of soils and plants was made by laboratory standards of the Activation Laboratories, a certified laboratory (ISO/IEC 17025), while quality control of the other analysis was carried out by technical replicates, use of certified standard solutions and method reagent blank.

Soil–plant transfer and translocation coefficients were calculated. The translocation coefficient ([total shoots element]/[total roots element]) indicates the translocation capacity of an element from roots to shoots (Huang and Cunningham 1996), while soil–plant transfer coefficient ([total shoots element]/[total soil element]) characterises the accumulation behaviour, i.e. if the plants can be considered as accumulators (transfer coefficient > 1) or excluders/non-accumulators (transfer coefficient < 1) of an element (Brooks 1998).

### Plant physiological analysis

The physiological analyses were carried out in *C. monspeliensis* leaves, frozen in liquid nitrogen at the moment of collection and kept at − 80 °C in a deep freezer, in order to prevent changes in physiological composition.

The extraction of the pigments was carried out by maceration of leaf samples in acetone:Tris-HCl 100 mM (80:20). The concentrations of chlorophyll *a* (chl *a*), chlorophyll *b* (chl *b*), total chlorophyll (chl total), anthocyanins and carotenoids were assayed by spectrophotometry (microplate reader Sinergy HT, Biotec, Winooski, USA) at 537, 647, 663 and 470 nm, using the equations described by Sims and Gamon (2002) and then expressed in μmol g^−1^ fresh weight (Richardson et al. 2002).

Reduced (GSH) and oxidised (GSSG) glutathione were analysed colorimetrically by the 2-vinylpiridine method (Anderson et al. 1992). Absorbance was recorded at 412 nm. The percentage of reduction corresponds to the percentage of GSH in the total glutathione pool and is defined as GSH/(GSH + GSSG) × 100.

Ascorbic (AsA) and dehydroascorbic (DAsA) acids were assayed using a method adapted from Okamura (1980) by Carvalho and Amâncio (2002). Absorbance was recorded at 525 nm. Standard curves of AsA in the range of 10–60 mM were prepared in 5% metaphosphoric acid. The concentration of DAsA was calculated by subtracting the AsA concentration measured from the total ascorbate assayed.

Hydrogen peroxide production was determined using a fluorometric horseradish peroxidase (HRP) linked assay (Amplex Red assay kit, Invitrogen). Leaf material was ground over activated charcoal in the presence of liquid nitrogen (Creissen et al. 1999). Absorbance was measured at 570 nm.

Total protein was extracted using an extraction buffer composed of TRIS 0.2 M (pH 8) containing 2% SDS (sodium dodecyl sulphate), 5 mM EDTA (ethylenediaminetetraacetic acid), 5 mM MgCl_2_, 10% glycerol and 2% 2-mercaptoethanol. At the moment of extraction, 2 mg mL^−1^ PMSF (phenylmethylsulphonyl fluoride) and 3% PVPP (polyvinylpolypyrrolidone) were added. Activated charcoal was added to all the extracts and the supernatants were used for the assays. Total protein was quantified using Bradford’s (1976) method with a commercial kit (Bio-Rad, Hercules, CA) according to the manufacturer’s instructions.

The enzyme activities were analysed using ca. 14 μg of protein in the case of ascorbate peroxidase (APX), monodehydroascorbate reductase (MDHAR) and superoxide dismutase (SOD) and ca. 35 μg of protein for glutathione reductase (GR), dehydroascorbate reductase (DHAR) and catalase (CAT). The activity of APX (EC 1.11.1.11) was assayed using a modified method of Hossain and Asada (1984). The reaction mixture contained 50 mM potassium phosphate–KOH (pH 7.5), 0.625 mM AsA and 0.125 mM EDTA. The oxidation rate of AsA was followed at 290 nm for 60 s after starting the reaction by adding of 0.2 mM H_2_O_2_ (ε_290_ = 2.8 mM^−1^ cm^−1^).

The activity of MDHAR (EC 1.6.5.4) was assayed by reduction of the absorbance at 340 nm due to the oxidation of NADH (ε_340_ = 6.22 mM^−1^ cm^−1^) (Arrigoni et al. 1981). The reaction mixture contained 50 mM Tris-HCl (pH 7.5), 0.2 mM NADH and 2.5 mM AsA. This reaction generates MDAsA by the ascorbate/ascorbate oxidase system (Arrigoni et al. 1981). To assay MDHAR activity, the rate of MDAsA-independent NADH oxidation (without AsA and ascorbate oxidase) was subtracted from the initial MDAsA dependent NADH oxidation rate (with AsA and ascorbate oxidase).

The determination of the activity of DHAR (EC 1.8.5.1) was based on the increase of the absorbance at 265 nm due to the formation of ascorbate (ε_265_ = 14 mM^−1^ cm^−1^) (Dalton et al. 1993). The reaction mixture contained 0.1 M Hepes-KOH buffer (pH 7.0), 2.5 mM GSH, 0.5 mM DAsA and 0.1 mM EDTA. The reaction rate was corrected for the non-enzymatic reduction of DAsA by GSH. A correction factor of 0.98 was applied in the assessment of enzyme activity to compensate for GSSG absorbance (Hernández-Jiménez et al. 2002; Redondo et al. 2009).

The determination of the activity of GR (EC 1.6.4.2) was based on the reduction of the absorbance at 340 nm due to the oxidation of NADPH (ε_340_ = 6.22 mM^−1^ cm^−1^) (Schaedle and Bassham 1977). The reaction mixture contained 50 mM Tris-HCl (pH 7.5), 0.15 mM NADPH, 0.5 mM GSSG and 3 mM MgCl_2_.

The activity of SOD (EC 1.15.1.1) was assayed at 550 nm using the ferrocytochrome c method and xanthine/xanthine oxidase as sources of superoxide radicals (McCord and Fridovich 1969). The reaction mixture contained 50 mM potassium phosphate–KOH buffer (pH 7.6), 0.1 mM EDTA, 0.01 mM cytochrome c, 0.05 mM xanthine and 0.03 units of xanthine oxidase.

The activity of CAT (EC 1.11.1.6) was assayed directly by the decomposition of H_2_O_2_ at 240 nm in a reaction mixture containing 50 mM potassium phosphate–KOH buffer (pH 7.0) and 10 mM H_2_O_2_ (Carvalho et al. 2006).

### Statistical analysis

The data obtained were analysed with the statistical program IBM-SPSS Statistics 23 for Windows. Data were checked for normality (Shapiro–Wilk test) and homogeneity of variances (Levene test) and, when possible, a simple ANOVA and Tukey test (*p* < 0.05) was applied. Data not satisfying these assumptions were analysed using a non-parametric analysis of Kruskal–Wallis test (*p* < 0.05) and the Man-Whitney *U* Test for comparison among areas. Principal component analysis (PCA) was applied to the data set for identifying the possible relations among chemical properties of the soils, multielemental concentrations in roots and shoots and in the available fraction of the soil, and multielemental concentrations in shoots and physiological parameters. For statistical purposes, the results below the detection limit were assumed as half of the detection limit.

## Results and discussion

### Chemical soil characteristics

Chemical characteristics of the soils are shown in Table 1. Mine soils were developed on heterogeneous mixtures of host rocks, influenced by acid mine drainage and/or different waste materials, which consequently influenced the characteristics of the soils. Due to this heterogeneity of the materials, chemical characteristics of soils from mining areas presented, in general, a wide range of values.

**Table 1:** Chemical leftacteristics of soils from São Domingos and Lousal mines (contaminated areas) and Pomarão (reference area)

	São Domingos			Lousal			Pomarão		
	Minimum	Maximum	Average	Minimum	Maximum	Average	Minimum	Maximum	Average
pH_H2O_	3.76	4.49	4.12b	4.09	5.40	4.55b	6.08	6.32	6.29a
Organic C (g kg^−1^)	5.90	22.4	15.1a	7.80	27.3	14.1a	6.90	14.90	10.7a
Total N (g kg^−1^)	0.47	1.93	1.10a	0.73	1.69	1.02a	0.34	0.75	0.56a
Extractable P (mg kg^−1^)	0.34	2.52	1.18b	1.35	2.49	2.00b	7.30	10.50	9.00a
Extractable K (mg kg^−1^)	45.7	90.9	69.9a	23.3	101	55.2a	70.0	138	95.0a
Electrical conductivity (μS cm^−1^)	130	520	235a	152	1191	495a	271	321	301a
Element	Total (mg kg^−1^)								
As	711	3030	1662a	62	662	460b	18	19	19c
Cd	0.3	1.3	1.0a	0.3	1.2	0.7a	0.3	0.3	0.3a
Cr	72	91	81a	71	128	106a	77	113	91a
Cu	203	342	253a	79	526	325a	25	47	32b
Mn	100	575	327b	500	1060	690ab	713	898	813a
Ni	10	48	34a	45	55	50a	31	42	35a
Pb	666	9210	3489a	95	2280	961a	28	50	39b
Sb	55	496	163a	21	189	74a	1.5	2.2	1.8b
Zn	36	186	129b	166	878	456a	92	123	104b
Element	Available fraction (mg kg^−1^)								
As	0.15	1.89	0.97a	0.03	0.45	0.26a	0.03	0.09	0.05a
Cd	0.01	0.07	0.04a	0.02	0.05	0.03a	0.01	0.03	0.02a
Cr	0.02	0.05	0.03a	0.03	0.07	0.05a	0.03	0.05	0.04a
Cu	2.68	9.95	5.11a	0.76	9.26	5.31a	0.09	2.16	0.78b
Mn	1.84	46.8	14.7b	26.1	49.3	46.5a	40.7	50.0	44.7a
Ni	0.06	0.39	0.27a	0.24	0.64	0.39a	0.08	0.29	0.15a
Pb	0.23	4.04	1.66a	0.05	0.98	0.51a	0.07	0.65	0.27a
Sb	0.03	0.22	0.14a	0.02	0.24	0.09a	< 0.01	0.01	0.01b
Zn	2.04	7.36	4.97a	5.90	15.8	10.8a	0.50	7.10	2.73a

Average data followed by a different letter indicates significance differences among areas (*p* < 0.05)

The pH values of the soils from both mine areas are very acid-to-acid due to mine wastes from which they were developed. These pH values were significantly lower than those from Pomarão. Independently of the studied area, no significant differences were found among electrical conductivities as well as the concentrations of total N, organic C and extractable K. However, concentrations of extractable P in soils from São Domingos and Lousal mines were lower than in soils collected in Pomarão (Table 1).

The soils from São Domingos and Lousal mine had very high total concentrations of As (only São Domingos), Cu, Pb and Sb, which are in contrast with the total concentrations of the same elements in the soils collected in Pomarão. Besides, the highest total concentration of Zn was obtained in Lousal soils while the highest total concentrations of Mn were found in soils from Pomarão. No significant differences were observed between the concentrations of Cr, Ni and Cd in the different studied areas (Table 1).

According to different reference guidelines for metal(loid) levels in soils (CCME 2007; VROM 2009), the total concentrations of As, Sb, Cu, Cr, Pb and Sb in soils from both mine areas (Table 1) exceeded the intervention values and maximum permitted levels for the protection of ecosystems and human health as well as commercial and industrial land use. The total concentrations of metal(loid)s in the soils from Pomarão (reference area) did not exceed those levels, except for As and Cr (CCME 2007). Nonetheless, the concentrations of these elements are within the range of values for non-contaminated soils from region and developed on the same geological substratum (Abreu et al. 2008, 2012b; Santos et al. 2012; Tavares et al. 2008).

Although the total concentrations of the elements in the mine soils were higher, compared to those in Pomarão soils, the element concentrations in the available fraction of the soils were low (< 13.3% of the total concentrations) independently of the studied area. Moreover, no significant differences in the concentrations of As, Cd, Cr, Ni, Pb and Zn in the available fraction were obtained among the three studied areas, although some soils from São Domingos and Lousal can reach higher concentrations compared to Pomarão soils (Table 1). The concentrations of Cu and Sb in the available fractions of the mine soils were significantly higher than those in Pomarão soils. Besides, Mn concentrations in the available fraction of soils from Pomarão and Lousal were significantly higher than those in soils of São Domingos (Table 1).

**Fig. 1:**
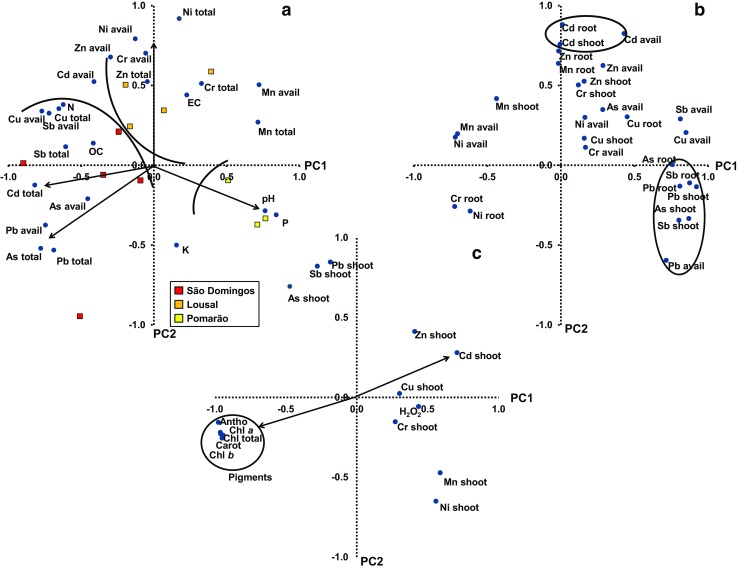
Principal components analysis and plots of **a** total and available metal(loid) concentrations and soil characteristics from the three studied areas; **b** metal(loid) concentrations in roots and shoots of *C. monspeliensis* and metal(loid) concentrations in the available fraction of soils from the three studied areas; and **c** metal(loid) concentrations in shoots of *C. monspeliensis* and pigments. Arrows and circles indicate and group the most relevant results of the PCA explained in the text. Element total: metal(loid) total concentration; element avail: metal(loid) available concentration; element shoot: metal(loid) shoot concentration; element root: metal(loid) root concentration; EC: electrical conductivity; OC: organic C; N: total N; K: extractable K; P: extractable P; Chl *a*: chlorophyll *a*; Chl *b*: chlorophyll *b*; Chl total: total chlorophyll; Antho: anthocyanins; Carot: carotenoids

The analysis of the PCA for soil characteristics (Fig. 1a) led to a reduction of the initial dimension of the dataset to two components, which explain 55.2% of the data variation (PC1 22.7%; and PC2 32.49% of the variance). The PC1 indicates that pH affects negatively the available contents of Cu, Sb and Pb in soils while available concentrations of Mn and Ni can be related to their total concentrations. Through PCA analysis, it was possible to obtain a clear separation of the studied areas. Thus, the soils from Pomarão, with high values of pH and extractable P contents as well as low concentrations of As, Cd and Ni in the total fraction and Cu, Cr, Ni, Pb and Sb in the available fraction, are differentiated from the mine soils, which have opposite characteristics. Within soil mines, Lousal soils are grouped especially by their high total concentrations of Ni and the concentrations of Cr and Ni in the available fraction, while São Domingos soils are distinguished mainly by their high total concentrations of As, Cd and Pb in the total fraction and the concentrations of As and Pb in the available fraction.

In general, concentrations of metal(loid)s in the total and available fractions as well as other chemical properties of the soils are in agreement with the range of values obtained in previous studies performed in the same areas (Abreu et al. 2008, 2012a, b; Alvarenga et al. 2012; Batista et al. 2017; Ferreira da Silva et al. 2005; Freitas et al. 2004; Pérez-López et al. 2014; Santos et al. 2012, 2014, 2016c).

### Concentrations of metal(loid)s in plants

The concentrations of metal(loid)s in shoots and roots of *C. monspeliensis* are shown in Table 2. Independently of the area, the concentrations of the elements in roots and shoots were higher than the concentrations of the same elements in the available fraction of the soils (Table 1), except for Sb in roots. In general, the concentrations of metal(loid)s in shoots and roots in *C. monspeliensis* growing in both mines showed a great heterogeneity, as also observed for other *Cistus* species growing in mining areas from the IPB (e.g. Abreu et al. 2012a, b; Santos et al. 2012, 2014 and references therein).

**Table 2:** Metal(loid) concentrations (mg kg^−1^) in roots and shoots of *C. monspeliensis* collected in São Domingos and Lousal mines (contaminated areas) and Pomarão (reference area)

Element	São Domingos			Lousal			Pomarão			Literature values
	Minimum	Maximum	Average	Minimum	Maximum	Average	Minimum	Maximum	Average	
Roots (mg kg^−1^)										
As	8.31	13.6	11.4a	0.84	3.70	2.29b	0.50	0.78	0.66b	–
Cd	0.29	2.21	1.32a	0.70	1.73	1.24a	1.02	1.32	1.16a	–
Cr	0.79	1.30	0.97c	0.94	3.22	1.90b	5.25	8.97	6.86a	–
Cu	17.4	34.6	26.7a	4.33	13.0	9.32b	9.52	13.7	11.2b	–
Mn	192	1963	764a	144	728	401a	478	769	575a	–
Ni	0.88	5.62	3.08b	0.94	2.12	1.54b	5.15	6.56	5.83a	–
Pb	3.74	90.7	35.0a	1.83	28.9	10.1a	4.78	9.34	7.72a	–
Sb	0.03	0.38	0.14a	0.02	0.15	0.06ab	0.01	0.02	0.01b	–
Zn	48.1	118	91.0a	64.3	187	117a	74.9	98.9	90.6a	–
Shoots (mg kg^−1^)										
As	2.22	75.8	29.7a	0.55	5.34	2.63b	1.06	1.22	1.05b	7.83^(1)^
										1.3–2.1^(2)^
										10–71^(3)^
Cd	0.37	2.57	1.56b	2.48	4.68	3.45a	1.39	1.90	1.45b	–
Cr	0.59	4.86	3.14a	1.22	2.02	1.40b	2.43	2.57	2.38a	–
Cu	8.34	46.1	26.8a	7.60	16.9	13.8a	28.7	30.4	28.6a	42.9 ± 0.79^(1)^
										5.2–16.0^(2)^
										27–80^(3)^
Mn	200	1991	1165ab	174	1387	815b	1721	1991	1828a	29.2 ± 5.1^(1)^
										1009–1045^(3)^
Ni	1.24	5.62	4.02b	2.41	6.36	4.14b	7.10	8.21	8.20a	0.72^(1)^
										3.3–5.9^(2)^
Pb	4.53	35.8	15.6a	2.54	9.76	6.13a	2.85	3.19	2.83a	9.14 ± 2.79^(1)^
										20.0–20.7^(2)^
										15–23^(3)^
Sb	0.04	0.87	0.25a	0.01	0.22	0.10a	0.06	0.08	0.07a	–
Zn	153	308	217b	259	531	408a	157	169	151c	319 ± 185^(1)^
										142.3–343.2^(2)^
										328–357 ^(3)^

Values found in the literature for *C. monspeliensis*: ^(1)^de la Fuente et al. (2010), shoots from Rio Tinto mining area; ^(2)^Freitas et al. (2004), leaves and twigs from São Domingos mine; ^(3)^Batista et al. (2017), leaves from São Domingos mine. For each element, average data followed by a different letter indicates significance differences among populations (*p* < 0.05)

*Cistus monspeliensis* from Pomarão showed the highest concentrations of Cr and Ni in roots and Ni, Cr and Mn in shoots. However, concentrations of As, Cu, and Sb in roots and As and Zn in shoots from São Domingos and Cd and Zn in shoots from Lousal were higher than those in Pomarão (reference area). Similar behaviour was observed in other species growing in contaminated and non-contaminated areas from the IPB, as *Cistus ladanifer* L. (As and Zn in shoots), *Cistus salviifolius* L. (e.g. As and Sb in shoots and roots) and *Lavandula pedunculata* (Mill.) Cav. (Abreu et al. 2012a; Santos et al. 2012, 2016c; Trigueros et al. 2012), as well as in *Erica andevalensis* (Cabezudo & J. Rivera) and *Erica australis* L. (Abreu et al. 2008; Pérez-López et al. 2014).

The PCA analysis (Fig. 1b) done to assess the possible relationship between the concentrations of metal(loid)s in the soil available fraction, and roots and shoots of *C. monspeliensis* can explain 52.9% of the data variation. The PC1, which explains 33.0% of the variance, shows that the concentrations of Pb and Sb in roots and shoots can be explained by the concentrations of the same elements in the available fraction of the soils. The same was obtained for Cd in PC2, which explains 19.9% of the variance. Also, PC2 shows a possible synergistic interaction Cd–Zn as reported by Kabata-Pendias (2011).

Intra- and inter-population differences were observed in the translocation behaviour (Table 3) of the elements in the plants. In general, plants from the three populations mainly translocated As, Cd, Cu, Mn, Ni, Sb and Zn from roots to shoots (Translocation coefficient > 1). This translocation behaviour differ to other species of the genus *Cistus,* such as *C. populifolius*, *C. salviifolius and C. ladanifer*, which mainly accumulated metal(loid)s in roots (Abreu et al. 2012a, b; Alvarenga et al. 2004; Santos et al. 2014). However, in general, the concentrations of the studied elements in *C. monspeliensis* shoots from the three populations were below the toxicity limit and/or within the range considered sufficient/normal for plants, except for As in plants from São Domingos, and Mn and Zn in plants from the three areas (Table 2) which present values considered as phytotoxic (Kabata-Pendias 2011). Despite these concentrations, no visual symptoms of toxicity were observed (data not shown). Moreover, an additional important aspect is that elemental concentrations in the shoots were below the toxicity limits for domestic animals (NRC 2005) and did not represent any environmental risk.

**Table 3:** Metal(loid) translocation from roots to shoots and metal(loid) soil–plant transfer coefficients of *C. monspeliensis* collected in São Domingos and Lousal mines (contaminated areas) and Pomarão (reference area)

Element	São Domingos			Lousal			Pomarão		
	Minimum	Maximum	Median	Minimum	Maximum	Median	Minimum	Maximum	Median
Translocation coefficient									
As	0.20	6.09	2.13	0.65	1.44	0.95	1.10	2.12	1.77
Cd	0.82	2.21	1.28	2.06	4.39	2.78	1.04	1.44	1.22
Cr	1.67	5.23	3.26	0.37	1.26	0.93	0.27	0.49	0.34
Cu	0.33	1.65	1.19	1.23	1.76	1.58	1.95	3.19	2.75
Mn	0.89	2.26	2.18	1.20	3.25	1.58	2.31	4.16	3.60
Ni	0.22	2.41	2.02	2.10	3.07	2.78	1.25	1.61	1.38
Pb	0.30	1.21	0.60	0.31	1.73	1.15	0.27	0.60	0.34
Sb	0.57	2.29	1.57	0.50	2.33	1.49	3.00	8.00	6.00
Zn	1.68	3.92	2.61	2.82	5.44	3.44	1.30	2.09	1.72
Soil–plant transfer coefficient									
As	0.01	0.04	0.02	< 0.01	0.01	0.01	0.05	0.07	0.06
Cd	0.28	4.83	2.07	3.10	10.23	4.92	3.53	6.33	4.63
Cr	0.02	0.07	0.05	0.01	0.02	0.02	0.02	0.03	0.03
Cu	0.04	0.23	0.08	0.03	0.10	0.05	0.57	1.22	1.15
Mn	2.00	5.19	3.29	0.29	2.31	1.03	1.97	2.79	2.08
Ni	0.05	0.17	0.12	0.05	0.12	0.09	0.22	0.26	0.22
Pb	0.00	0.02	0.01	< 0.01	0.03	0.02	0.06	0.09	0.08
Sb	< 0.01	< 0.01	< 0.01	< 0.01	0.01	0.01	0.03	0.05	0.03
Zn	1.05	4.25	1.60	0.54	1.56	1.16	1.34	1.71	1.37

Otherwise, plants from Pomarão mainly stored Cr and Pb in roots (Translocation coefficient < 1). The storage in roots and/or decrease of the translocation of the potentially hazardous elements from roots to shoots can be considered a tolerance mechanism (Abreu et al. 2014; Hossain et al. 2012).

Taking into account the few published studies on the concentrations of potentially toxic elements in *C. monspeliensis* (Batista et al. 2017; De la Fuente et al. 2010; Freitas et al. 2004) (Table 2), *C. monspeliensis* shoots present a wide range of element concentrations. Nonetheless, most of the element concentrations obtained in the present study for *C. monspeliensis* are in the same range than for other species of the genus *Cistus* (e.g. As in *C. salviifolius* shoots from São Domingos, Cu in *C. ladanifer* roots from Lousal) growing in the same mine areas (Abreu et al. 2012a, b; Freitas et al. 2004; Santos et al. 2009, 2012, 2014).

Concerning the plant accumulation behaviour, evaluated by the soil–plant transfer coefficient (Table 3), plants from the three populations were Zn, Mn and Cd accumulators but not hyperaccumulators. For the other studied elements and independently of the population, the plants can be considered non-accumulators.

### Concentration of pigments in leaves

**Fig. 2:**
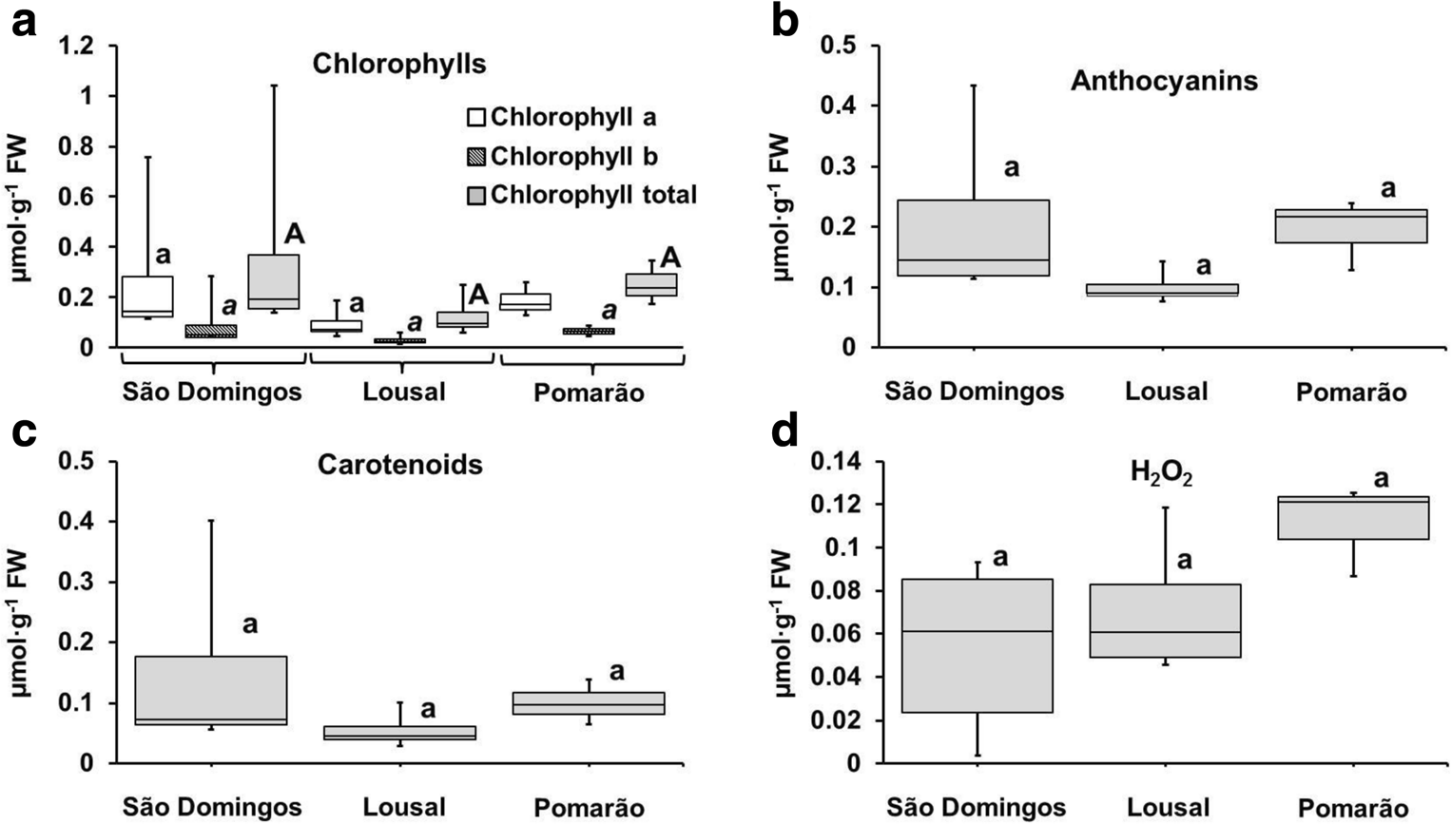
**a** Chlorophyll (total, *a* and *b*); **b** anthocyanins; **c** carotenoids; and **d** hydrogen peroxide (H_2_O_2_) contents in shoots of *C. monspeliensis* in each studied area. Box plot data distribution (min, Q1, median, Q3, max) for each parameter is indicated for each studied area. Black bars show the maximum and minimum values in each studied populations. Values with same letter (lowercase letter, lowercase italic or uppercase letter) indicate that there are no significant differences for each parameter among studied areas (*p* < 0.05)

Pigment concentrations in the leaves of *C. monspeliensis* are shown in Fig. 2a, b, c. In general, the excess of potentially hazardous elements in leaves can modify the concentration of pigments, which are usually associated to visual symptoms of plant disease and impaired photosynthetic activity (Kabata-Pendias 2011; Márquez-García and Córdoba 2009; Pang et al. 2003; Santos et al. 2016c; Tewari et al. 2008). However, independently of the population, no visual alteration in leaf colour was observed.

Although intra-population variation can be pointed out, no significant differences were obtained between the concentrations of chlorophylls (*a*, *b* and total), anthocyanins and carotenoids in the leaves from the three populations (Fig. 2a, b, c). Similar results were observed between contents of carotenoids in leaves of *E. australis*, *C. ladanifer* and *L. pedunculata* collected in different mining areas from IPB and in non-contaminated areas (Márquez-García and Córdoba 2009; Santos et al. 2013, 2016c).

A PCA was carried out to evaluate the possible influence of the contents of metal(loid)s on pigments in *C. monspeliensis* shoots (Fig. 1c), which was determined only for PC1 (43.99% of variance). The results showed that only Cd concentrations in shoots can affect negatively the concentrations of all studied pigments. Thus, the low contents of chlorophylls, anthocyanins and carotenoids in *C. monspeliensis* in the three studied areas might be attributed to the high level of solar radiation, air temperature and low humidity, stress factors associated to the Mediterranean conditions that occur in these areas (Correia 2002; Santos et al. 2013).

### Concentration of H_2_O_2_

Hydrogen peroxide content in the shoots of *C. monspeliensis* is shown in Fig. 2d. Plants under normal physiological conditions produce significant amounts of H_2_O_2_ as a by-product of their metabolism and, under various stress factors, namely high concentrations of metal(loid)s, H_2_O_2_ levels tend to increase due to its speed of formation exceed the capacity for scavenging (Caverzan et al. 2012). On the other hand, plants can eliminate H_2_O_2,_ through detoxification mechanisms, in order to limit the peroxidation reactions of the membrane lipids (Howlett and Avery 1997). The lowest levels of H_2_O_2_ in *C. monspeliensis* from mining areas, especially in some plants from São Domingos (Fig. 2d) can suggest the rapid elimination of this compound.

Comparing the studied populations, no significant differences were obtained due to the high variability of H_2_O_2_ concentrations in *C. monspeliensis*. Similar H_2_O_2_ concentrations were also reported in leaves of *E. australis* growing in mine wastes and uncontaminated soils from Spanish IPB (Márquez-García and Córdoba 2009). The PCA analysis indicates that this ecophysiological parameter is not explained by the concentrations of the studied metal(loid)s in the shoots.

### Antioxidative enzymes and antioxidant molecules

**Fig. 3:**
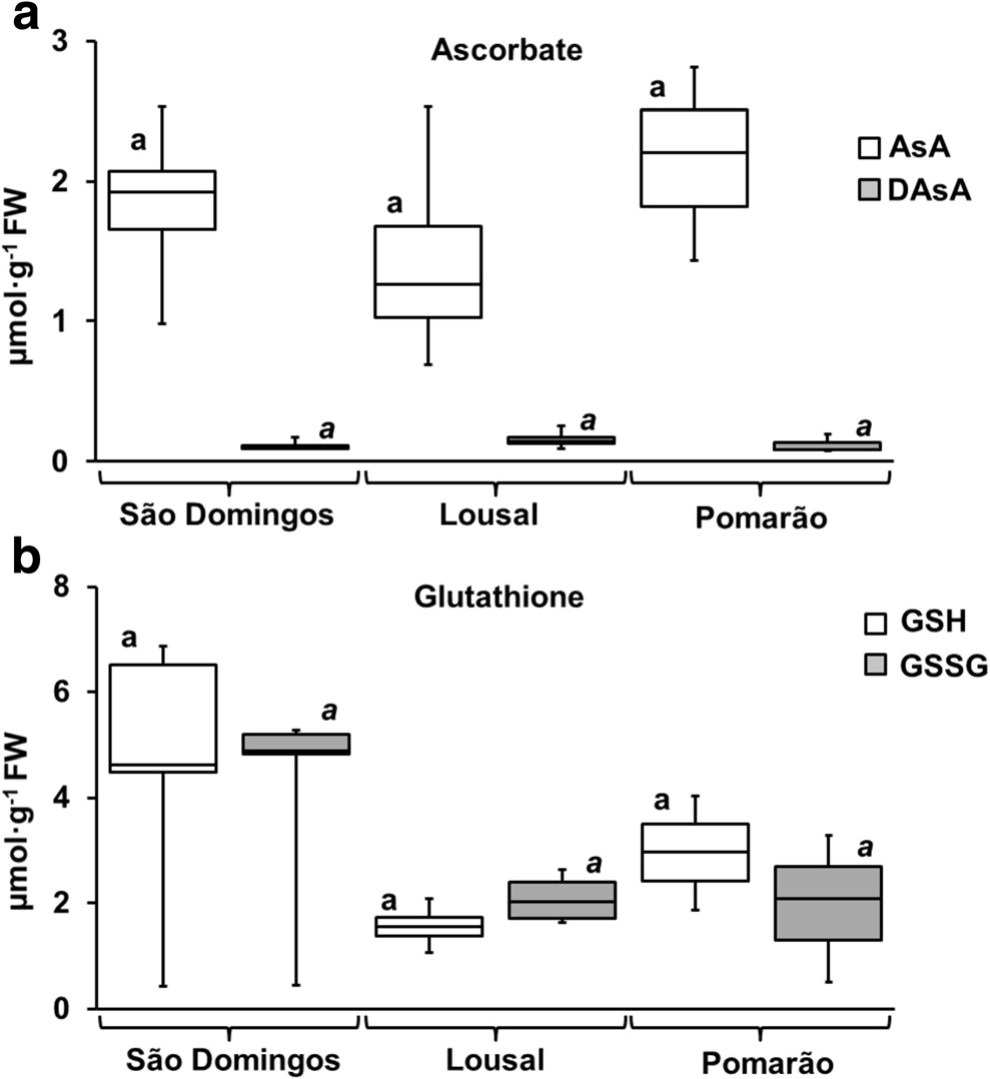
**a** Concentrations of reduced (AsA) and oxidised (DAsA) ascorbate and **b** reduced (GSH) and oxidised (GSSG) glutathione in shoots of *C. monspeliensis* from the different studied areas. Box plot data distribution (min, Q1, median, Q3, max) for each parameter is indicated for each studied area. Black bars show the maximum and minimum values in each studied populations. Values with same letter (lowercase letter, or lowercase italic) indicate that there are no significant differences for each parameter among studied areas (*p* < 0.05)

Ascorbate and glutathione contents in the leaves of *C. monspeliensis* are shown in Fig. 3. No significant differences in the concentrations of ascorbate and glutathione were obtained among plants of the three studied populations. Similar concentrations of glutathione in leaves of *P. lanceolata* and *C. arenosa* from contaminated and non-contaminated areas were also reported by Nadgórska-Socha et al. (2013).

When assessing the levels of reduced and oxidised ascorbate (AsA and DAsA, respectively) in leaves of *C. monspeliensis* from the three studied areas (Fig. 3), the reduction state was high in all cases. Generally, the maintaining of a high percentage of AsA is essential for the proper scavenging of ROS in cells (Mittler 2002), so the results obtained for ascorbate are a good indication of the cell’s redox state. The percentages of AsA reduction in the three populations was in the same range varying between 73.1 and 97.1%. Nevertheless, the reduction state of glutathione (GSH) was generally low and the only parameter significantly lower in plants from Lousal (39.5–46.1%) and São Domingos (47.2–57.2%) than in plants collected in Pomarão (58.8–78.5%). These results can indicate that the plants from mines can be under oxidative stress that impaired the normal functioning of the reduction cycle of glutathione.

**Fig. 4:**
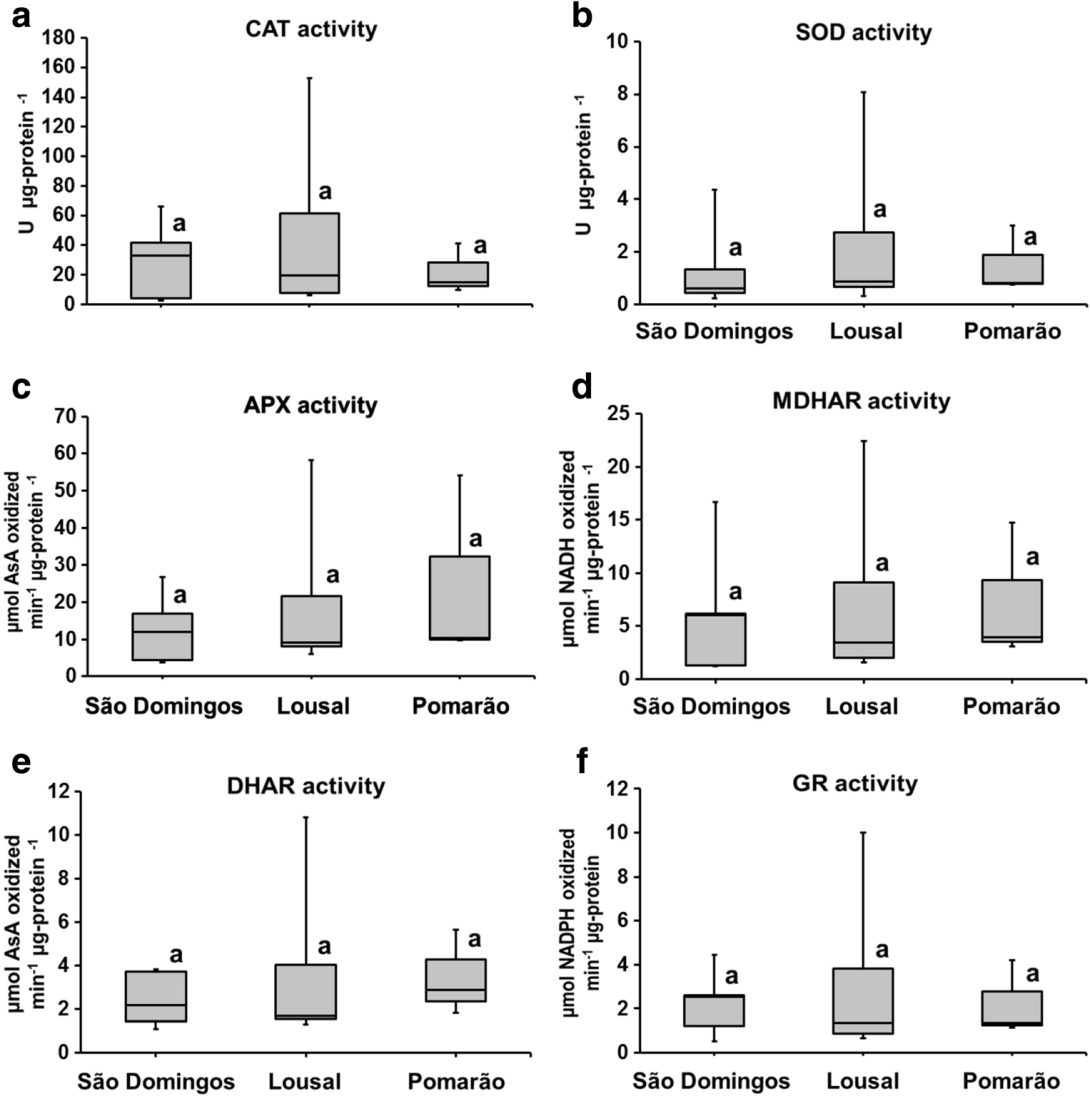
Total soluble enzyme activities in shoots of *C. monspeliensis* from the different studied areas: **a** catalase (CAT), **b** superoxide dismutase (SOD), **c** ascorbate peroxidase (APX), **d** monodehydroascorbate reductase (MDHAR), **e** dehydroascorbate reductase (DHAR), **f** glutathione reductase (GR). Box plot data distribution (min, Q1, median, Q3, max) for each parameter is indicated for each studied area. Black bars show the maximum and minimum values in each studied populations. Values with same letter indicate that there are no significant differences for each parameter among studied areas (*p* < 0.05)

Activities of antioxidative enzymes in the leaves of *C. monspeliensis* are shown in Fig. 4. In general, under oxidative stress, plants can also stimulate the activity of antioxidative enzymes, which remove and neutralise ROS (Pang et al. 2003; Santos et al. 2009). However, no significant differences in the antioxidative enzyme activities were obtained among the studied populations. These results suggest that *C. monspeliensis* plants from the three studied areas are able to adapt their enzyme activities and concentrations of antioxidant molecules to the concentrations of metal(loid)s in their shoots, showing high tolerance to these elements. Therefore, the potential toxicity caused by toxic elements did not trigger the activities of antioxidative enzymes. Similar activities of some antioxidative enzymes were also observed in *E. australis* (e.g. CAT and APX), *C. ladanifer* (e.g. SOD), *L. pedunculata* (e.g. SOD) and *P. lanceolata* (e.g. SOD) and *C. arenosa* (e.g. SOD) growing in soils affected and not affected by multielemental contamination of the mining activity (Márquez-García and Córdoba 2009; Nadgórska-Socha et al. 2013; Santos et al. 2009, 2016c).

## Conclusions

The soils from São Domingos and Lousal mining areas showed low values of pH and high total metal(loid)s concentrations, mainly As, Sb, Cu, Zn and Pb. However a clear separation (PCA) of the soils from the studied areas was obtained through pH, concentrations of extractable P, total concentrations of As, Cd and Ni and concentrations of Cu, Cr, Ni, Pb and Sb in the available fraction of the soils.

Only some soil parameters explained the availability of the elements in the soils, namely pH values (availability of Cu, Sb and Pb) and the total concentrations of Mn and Ni (availability of the same elements). In spite of the high total concentrations of the potentially hazardous elements in the mining soils, the concentrations of these elements in the available fraction were low and similar independently of the studied areas. This fact could explain the general tendency to the similar concentrations of the same elements in shoots and roots of *C. monspeliensis* growing in soils with different levels of multielemental contamination and in non-contaminated soils.

In general, *C. monspeliensis* from the three studied populations were accumulators of Zn, Cd and Mn but not hyperaccumulators. Although the majority of the metal(loid)s and nutrients were translocated from roots to shoots, only some elements reached phytotoxic concentrations in the shoots (As in shoots from São Domingos; Mn and Zn in some plants from the three populations).

Independently of the mine area and soil characteristics, *C. monspeliensis* colonised the contaminated soils showing great tolerance and adaptability to limiting conditions for plant growth and oxidative stress as shown by the ecophysiological parameters. Taking into account the metal(loid) concentrations in shoots, which were under the toxicity limits for domestic animals, and the lack of phytotoxicity symptoms, as well as the dense soil cover and considerable deep root system, *C. monspeliensis* have potential for natural soil rehabilitation or to be used in assisted soil recovery programs leading to revegetation of degraded and abandoned mine areas under Mediterranean conditions.
